# Supplementary material for: Origin of the Laurentian Great Lakes fish fauna through upward adaptive radiation cascade prior to the Last Glacial Maximum
Source: Commun Biol. 2024 Aug 12;7:978. doi: 10.1038/s42003-024-06503-z (PMC11319351; doi:10.1038/s42003-024-06503-z)
Supplement: Supplementary file 2 — Supplementary Information [file 42003_2024_6503_MOESM2_ESM.pdf]

Origin of the Laurentian Great Lakes fish fauna through upward adaptive radiation cascade prior to the Last Glacial Maximum

SUPPLEMENTARY INFORMATION

Supplementary Table 1 – Repetitive element classification of *Coregonus artedi* reference genome. Repetitive elements were identified and enumerated using RepeatModeler and RepeatMasker.

		Repeat family	Number of elements	Total length		Percentage of sequence
**Retroelements**			1130493	411860062	bp	16.53%
	**SINEs:**		33907	5379106	bp	0.22%
		Penelope	18327	4576819	bp	0.18%
	**LINEs:**		844892	267882416	bp	10.75%
		CRE/SLACS	1	39	bp	0.00%
		L2/CR1/Rex	644179	193073504	bp	7.75%
		R1/LOA/Jockey	27293	5842151	bp	0.23%
		R2/R4/NeSL	2735	759541	bp	0.03%
		RTE/Bov-B	37778	14984800	bp	0.60%
		L1/CIN4	39270	14757363	bp	0.59%
	**LTR elements:**		251694	138598540	bp	5.56%
		BEL/Pao	7060	4481693	bp	0.18%
		Ty1/Copia	4849	1200653	bp	0.05%
		Gypsy/DIRS1	147220	104647288	bp	4.20%
		Retroviral	69565	23232687	bp	0.93%
**DNA transposons**			1232835	405649087	bp	16.28%
		hobo-Activator	188884	43955586	bp	1.76%
		Tc1-IS630-Pogo	840873	316778428	bp	12.71%
		En-Spm	0	0		0.00%
		MuDR-IS905	0	0		0.00%
		PiggyBac	13274	3205489	bp	0.13%
		Tourist/Harbinger	16759	3945674	bp	0.16%
**Other**		(Mirage, P-element, Transib)	1898	377242	bp	0.02%
**Total**				1748616067	bp	70.17%

Supplementary Table 2 – Resequenced *Coregonus* sample collection locations and sequencing statistics. *Coregonus artedi* (CA), *C. hoyi* (CH), *C. kiyi* (CK) collected in Lake Superior, and *C. nigripinnis* (CN) samples were collected in Lake Nipigon. Resequencing statistics include total number of reads, average sequencing depth, breadth of the reference genome covered, the percentage of reads that aligned to the reference, and the percent of paired end reads that are correctly oriented to one another.

Sample	Latitude	Longitude	Read count	Average depth	Breadth of coverage%	Alignment%	Proper pair%
CA01	46.8062	-90.78238	216,228,23	13.16	95.62	99.74	95.88
CA02	46.9541	-90.47148	195,120,83	12.05	95.89	99.59	95.18
CA03	46.9541	-90.47148	215,793,79	13.12	95.67	99.73	96.10
CA04	46.9541	-90.47148	193,652,27	11.80	95.38	99.69	95.97
CH01	46.8920	-90.53256	198,414,32	12.02	95.71	99.72	95.38
CH02	46.8854	-91.21529	192,030,67	11.63	95.37	99.42	95.40
CH03	46.8541	-91.21529	195,800,07	11.92	95.24	99.64	95.73
CH04	46.8854	-91.21529	210,419,23	12.78	95.59	99.69	95.61
CK01	46.8920	-90.53256	193,206,74	11.74	95.35	99.62	95.42
CK02	47.4966	-89.99913	195,395,13	11.93	95.24	99.71	95.90
CK03	47.1571	-89.96871	201,485,81	12.24	95.58	99.67	95.52
CK04	47.4165	-88.46418	191,827,97	11.66	95.10	99.32	95.53
CN01	49.6760	-88.26014	255,236,14	15.38	95.83	99.76	94.93
CN02	49.6760	-88.26014	209,838,08	12.73	95.29	99.65	95.13

Supplementary Table 3 – *Salvelinus namaycush* genome resequencing statistics. Total number of Illumina reads, average sequencing depth, breadth of the reference genome covered, percentage of reads that aligned to the reference, and percent of paired-end reads that were correctly paired and oriented after mapping.

Sample	Morph	Read count	Average depth	Breadth of coverage%	Alignment%	Proper pair%
LS01	lean	248,817,553	20.45	97.68	97.19	92.77
LS02	lean	215,033,259	17.51	97.58	97.10	92.79
LS05	siscowet	212,805,221	17.12	97.53	97.22	92.81
LS07	lean	197,718,905	16.30	97.58	97.07	92.68
LS08	siscowet	206,829,006	16.78	97.41	97.32	92.98
LS09	siscowet	209,119,345	16.96	97.22	97.39	93.97

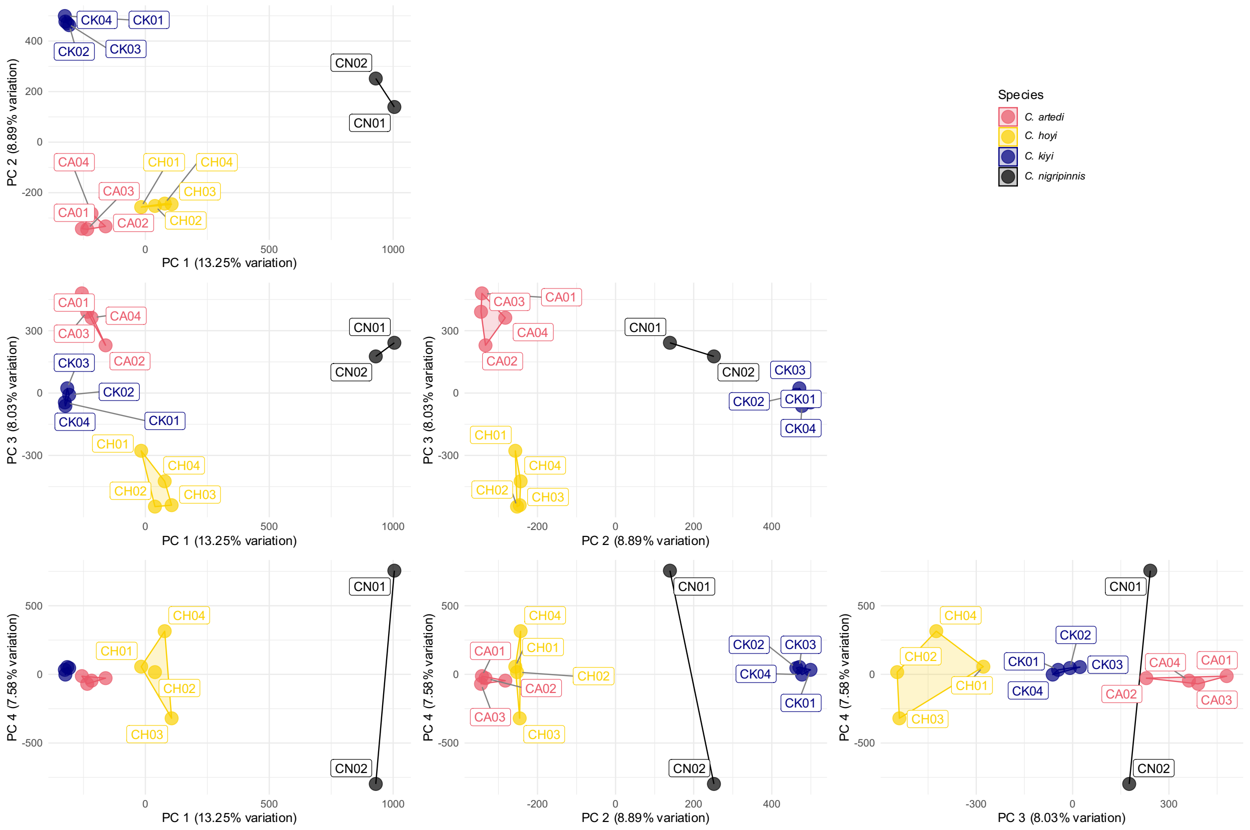

Supplementary Figure 1 – Laurentian Great Lakes *Coregonus* species population structure. Principal component analysis plots of 15,331,196 bi-allelic SNPs from *C. artedi*, *C. hoyi*, *C. kiyi*, and *C. nigripinnis*.

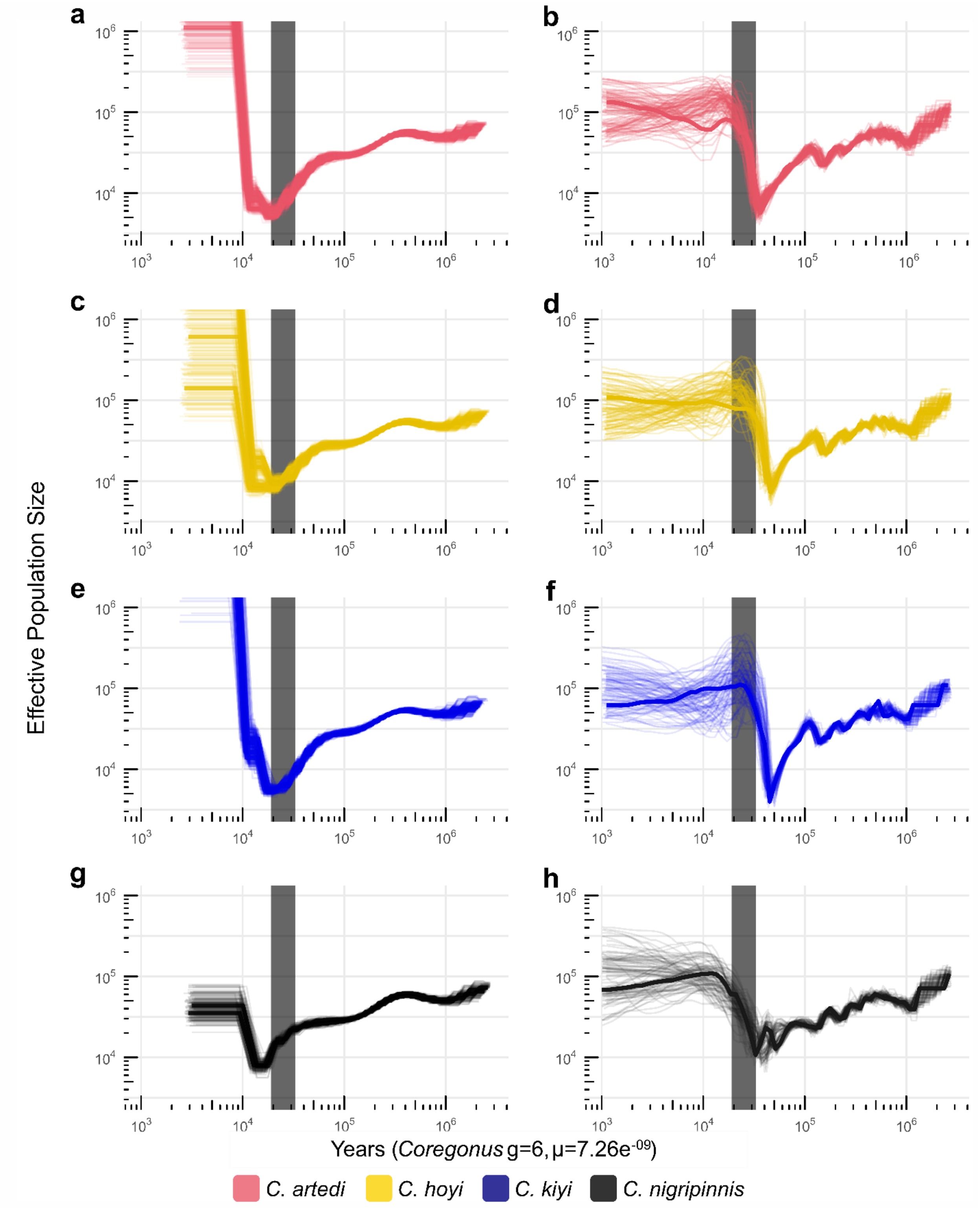

Supplementary Figure 2 – Inferred trajectories of Great Lakes *Coregonus* spp. effective population size through time. Results from PSMC (left column) and SMC++ (right column) for

Coregonus artedi (**a,b**), *C. hoyi* (**c,d**), *C. kiyi* (**e,f**), *C. nigripinnis* (**g,h**). Faint lines indicate bootstrap replicates (n=100). For each analysis, generation time was set to six years and the mutation rate was set to $7.26e^{-09}$. The dark grey bar represents the Last Glacial Maximum (19-33 ka).

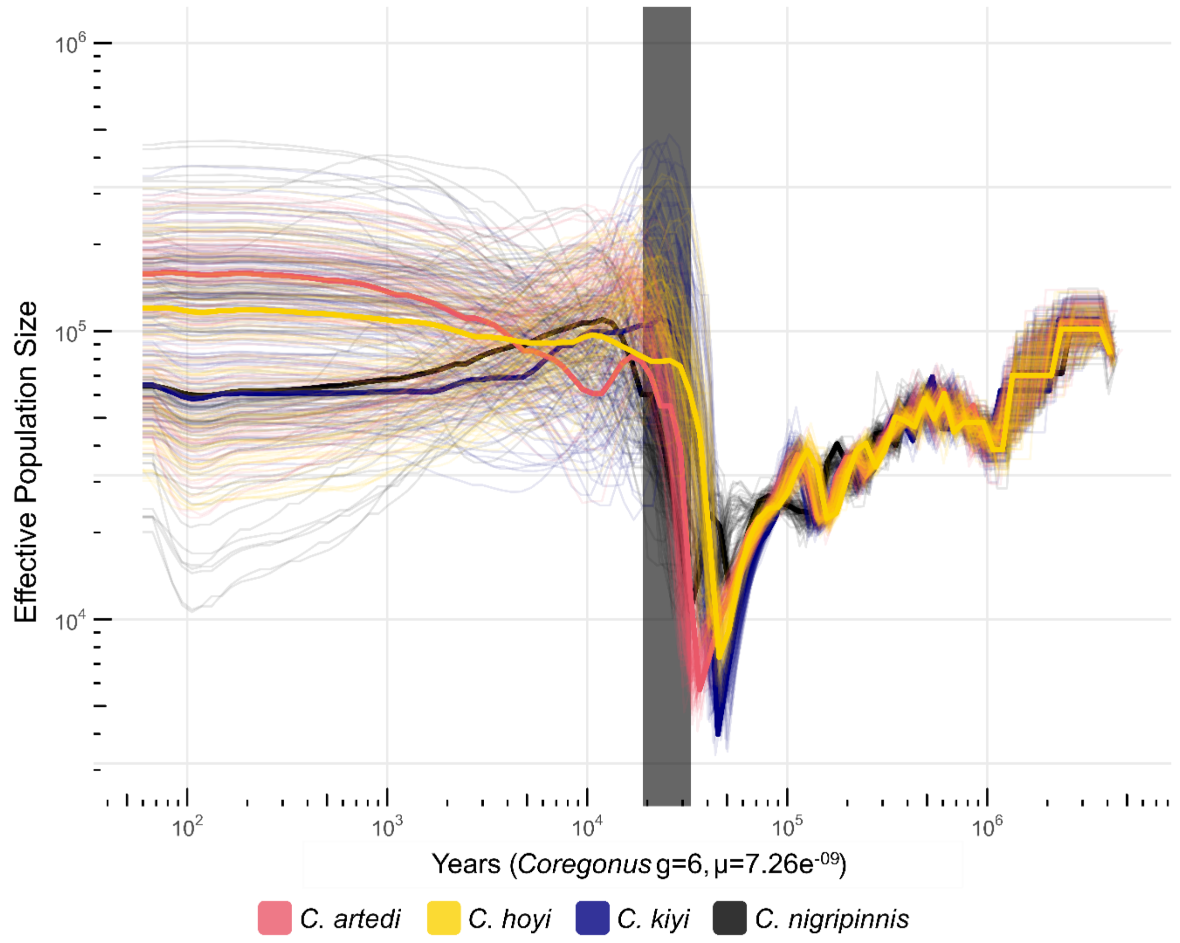

Supplementary Figure 3 – Estimated effective population size trajectories through time for four species of *Coregonus* (*C. artedi*, *C. hoyi*, *C. kiyi*, and *C. nigripinnis*). Estimates of variation for each sample were estimated by running SMC++ with 100 bootstrap replicates from 5 mb windows across the genome. Generation time for all samples was set to six years and the mutation rate (μ) to $7.26e^{-09}$ mutations per site per generation. The dark grey bar indicates the time range of the Last Glacial Maximum (19-33 ka).

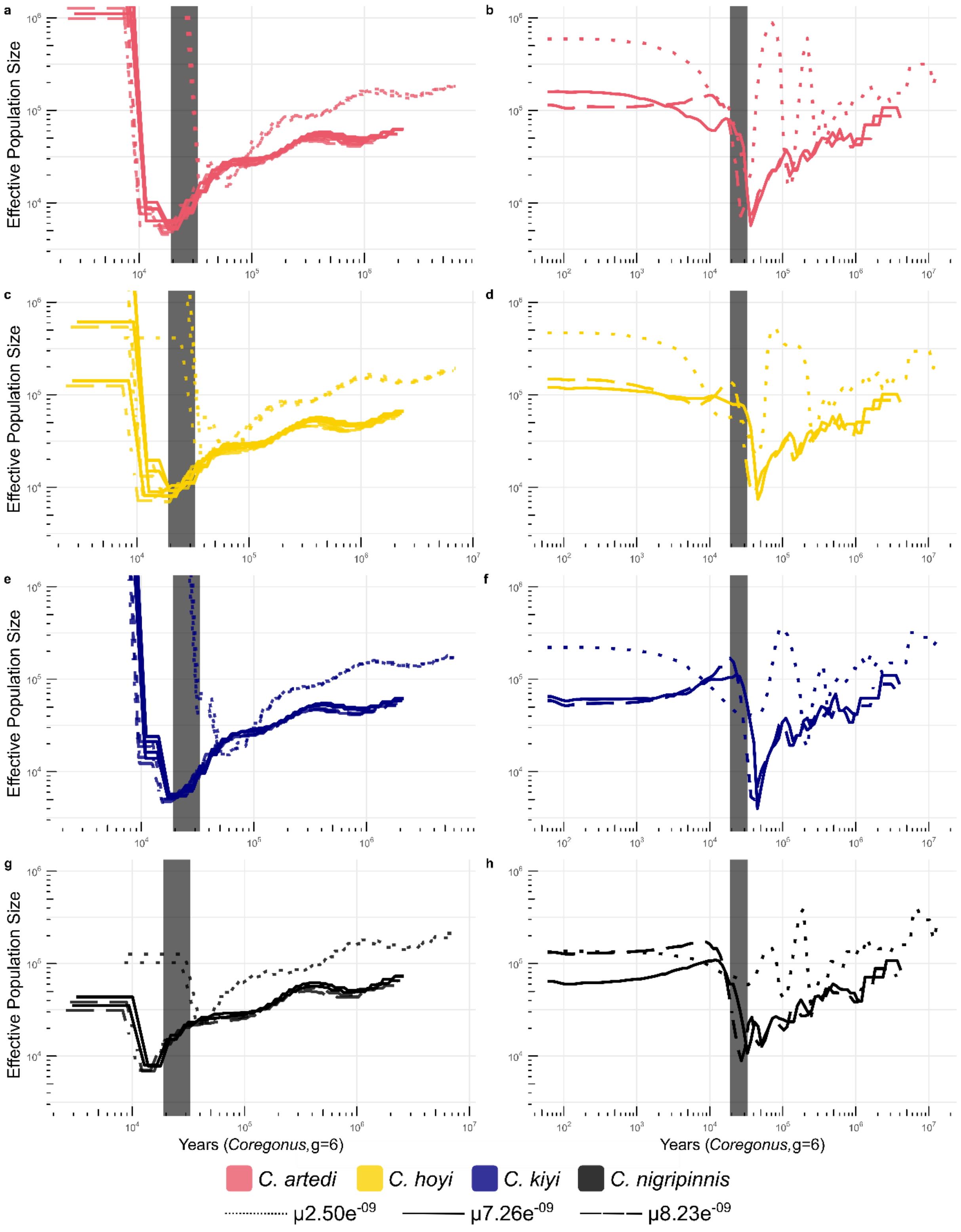

Supplementary Figure 4 – Variable mutation rate analyses. Estimates of historical demography with PSMC (left column) and SMC++(right column) for *Coregonus artedi* (**a,b**), *C.*

hoyi (**c,d**), *C. kiyi* (**e,f**), *C. nigripinnis* (**g,h**). For each analysis, the generation time was set to six years. Each sample was run with a mutation rate of $2.50e^{-09}$, $7.26e^{-09}$, and $8.23e^{-09}$. The dark grey bar represents the time period of the Last Glacial Maximum (19-33 ka).

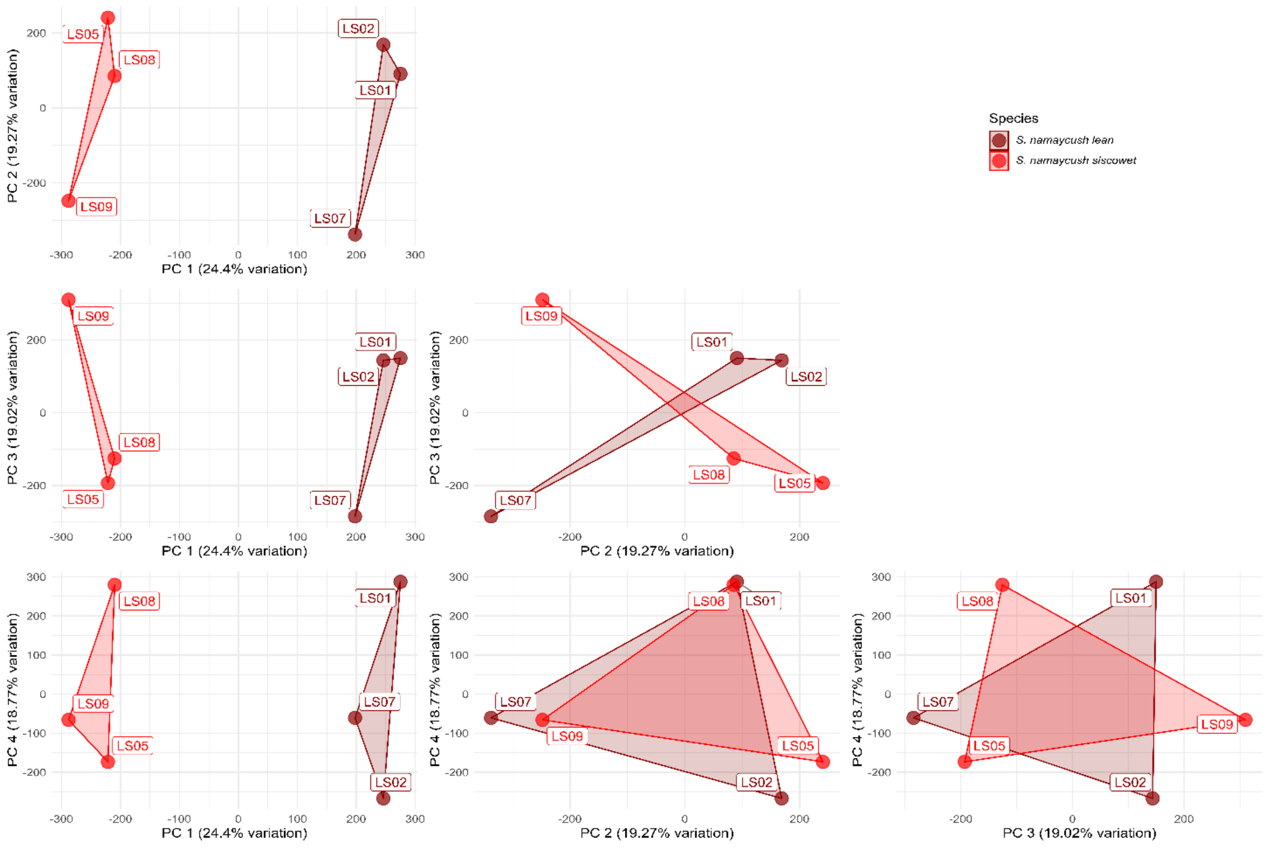

Supplementary Figure 5 – *Salvelinus namaycush* population structure. Principal component analysis of 3,534,068 bi-allelic SNPs from lean and siscowet morphs.

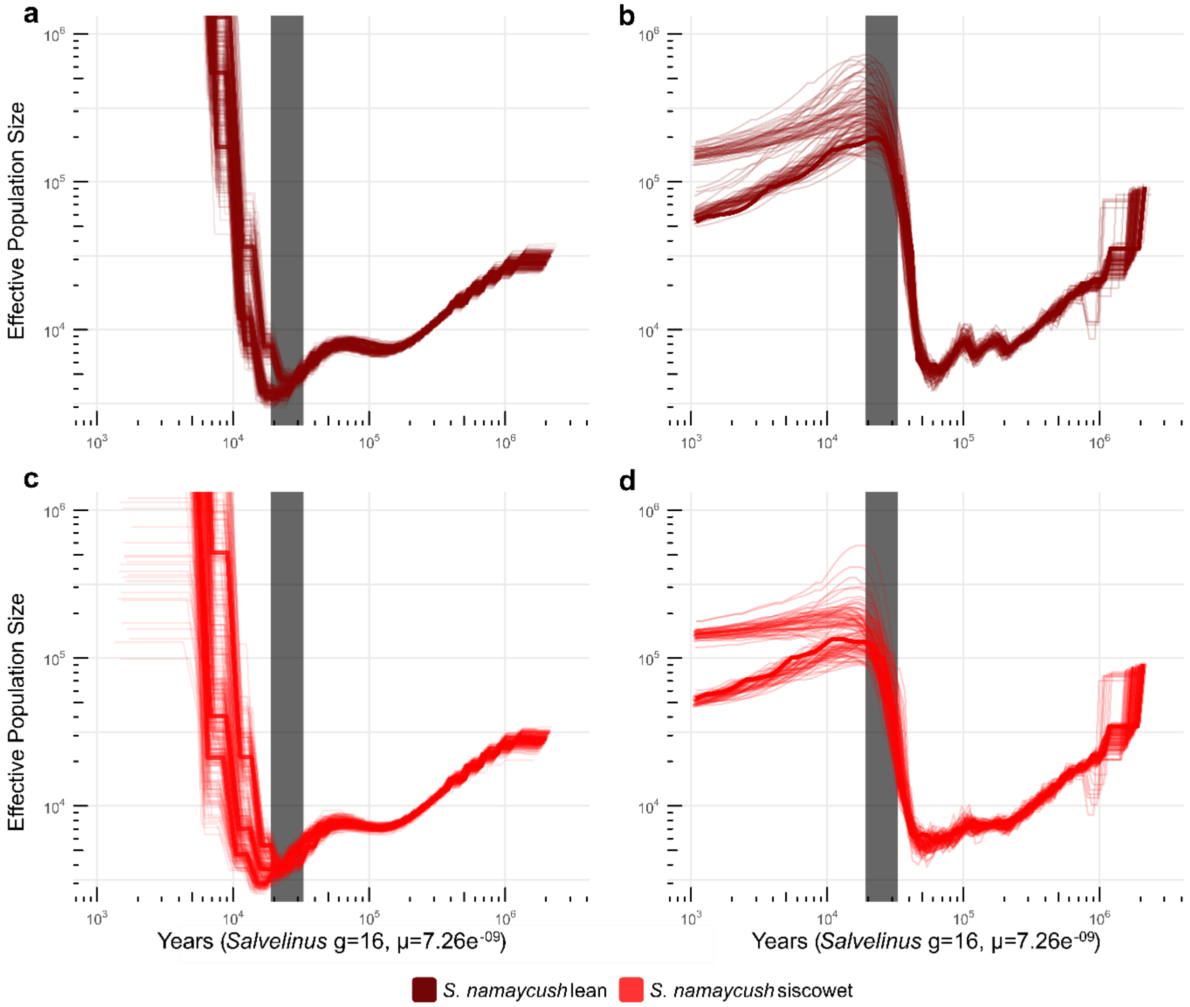

Supplementary Figure 6 – Effective population size estimates for *Salvelinus namaycush* morphs. Lean (**a,b**) and siscowet (**c,d**) morph samples with 100 bootstrap replicates for each sample produced by PSMC (**a,c**) and SMC++ (**b,d**). The dark grey bar represents the time range of the Last Glacial Maximum (19-33 ka).

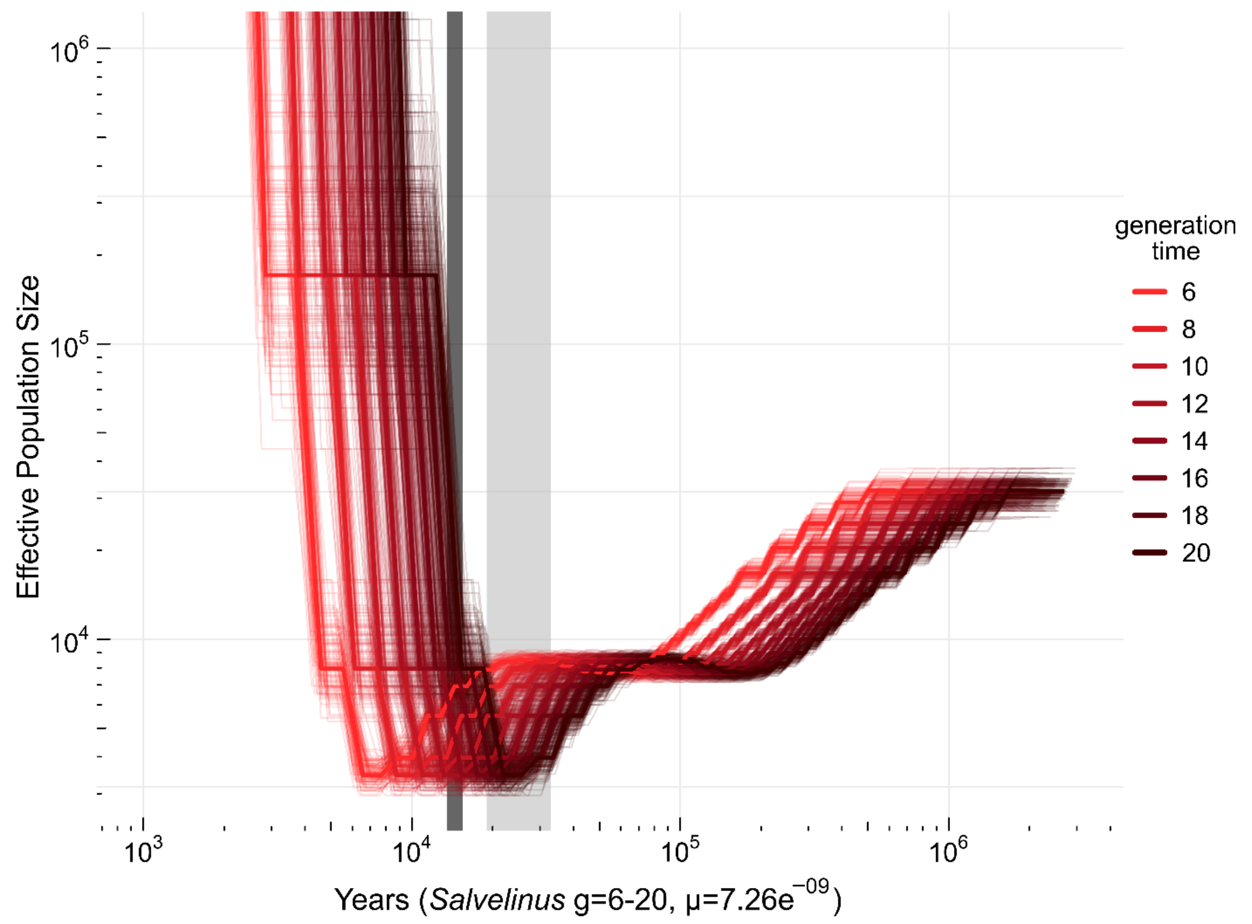

Supplementary Figure 7 – Generation time sensitivity plot. Estimated effective population size through time for one individual of the *S. namaycush* lean morph (LS01) from PSMC. Each set of lines indicates bootstrap replicates plotted with different generation times ranging from six to twenty years. Mutation rate parameters were set at a constant rate of 7.26e-09 mutations/site/generation. The light grey bar represents the time of the Last Glacial Maximum (19-33 ka), and the dark grey bar represents the time point when the Laurentide ice sheet was no longer covering the present-day Great Lakes (10.3 ka).

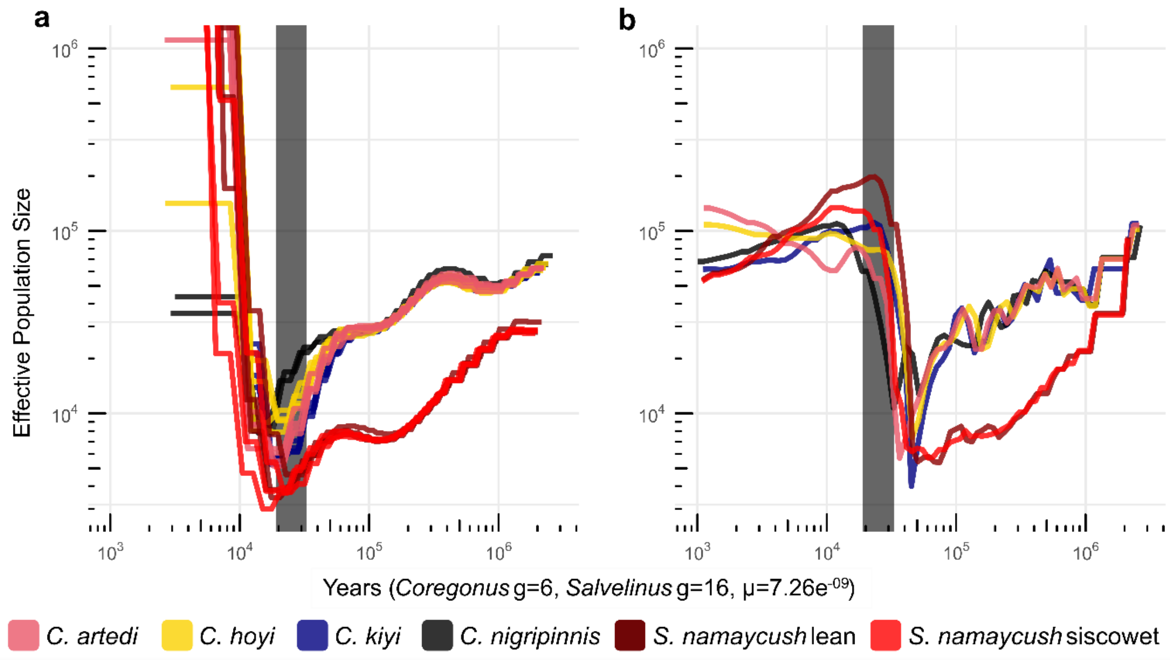

Supplementary Figure 8 – Effective population size estimates through time. (a) *Salvelinus namaycush* and *Coregonus* species estimates of effective population size using PSMC. **(b)** Estimated effective population sizes using SMC++. For both analyses the mutation rate was set at 7.26e^{-09} mutations per site per generation. Generation times for *Coregonus* species and *S. namaycush* were set at 6 years and 16 years respectively. The dark grey bar in both plots indicates the time range of the Last Glacial Maximum (19-33 ka).

SUPPLEMENTAL METHODS

Resequenced sample read statistics

All Illumina reads for the resequenced *Coregonus* samples were concatenated for each individual and read counts for were calculated with seqkit 'stats' (Shen, Le, Li, & Hu, 2016). Following mapping and variant calling the average depth of coverage was generated from alignment data files using samtools 'depth'. The percentage of the reference genome that had reads mapped to it (breadth of coverage) were calculated using samtools 'mpileup' using the alignment files. The alignment rate for all reads and proper pair reads for each individual were assessed with samtools 'flagstat'

Appendix I – Generation time estimates for Lake Superior coregonines

We used the method of Leslie[1] to estimate mean generation time (T) using age-specific elements in a life table: $T = \sum x l_x m_x / R_0$, where x = age in years, l_x = age-specific relative survivorship, m_x = age-specific fecundity, and $R_0 = \sum l_x m_x$.

We were able to acquire necessary information to model T for *C. artedi*, *C. hoyi*, and *C. kiyi*. To estimate relative survivorship by age, we used estimates of total instantaneous mortality rate (Z) provided by Pratt and Chong[2] for Lake Superior (*C. artedi* Z = 0.321; *C. hoyi* Z = 0.322; *C. kiyi* Z = 0.487). Estimates of age-specific fecundity for *C. artedi* were obtained from Yule et al.[3].

The development of life tables for *C. hoyi* and *C. kiyi* involved additional steps. We used von Bertalanffy growth equations for these species[2] which predicted fork length (FL) from age. We converted FL to total length (TL) based on measurements from digital images (*C. hoyi* TL/FL = 1.1011, N=45, length range = 83-310 mm TL; *C. kiyi* TL/FL = 1.095, N =37, length range = 9.4 – 25.9 mm). The *C. kiyi* total fecundity versus TL relationship was taken from Vinson et al.[4]. *C. hoyi* fecundity was obtained from Dryer and Beil[5]. These authors provided estimates of average *C. hoyi* fecundity in eight length bins, ranging from 21.3-29.7 mm TL (their Table 15). We developed a model predicting log (fecundity) from log (L) by weighting the bin estimates by sample size. The resulting model was as follows:

Log (Fecundity) = 2.7742 x Log (TL) – 0.1030: adjusted R^2 = 0.91).

Predictions of TL at age for *C. hoyi* were used to predict age-specific fecundity with this relationship.

Results

The estimates of mean generation time of *C. artedi* equaled 6.41 years, *C. hoyi* was 6.32 years, and *C. kiyi* 5.96 years.

Supplementary Table 4 - Life table for Lake Superior *C. artedi*.

Age (x)	Relative N (l_x)	Fecundity (m_x)	$l_x m_x$	$x l_x m_x$
3	1.0000	12,453	12,453	37,359
4	0.7254	15,797	11,460	45,838
5	0.5262	18,789	9,888	49,438
6	0.3817	21,429	8,180	49,083
7	0.2769	23,717	6,568	45,975
8	0.2009	25,653	5,153	41,227
9	0.1457	27,237	3,969	35,723
10	0.1057	28,469	3,010	30,096
11	0.0767	29,349	2,251	24,758
12	0.0556	29,877	1,662	19,945
13	0.0404	30,053	1,213	15,767
14	0.0293	29,877	875	12,245
15	0.0212	29,349	623	9,349
16	0.0154	28,469	439	7,017
17	0.0112	27,237	304	5,175
18	0.0081	25,653	208	3,744
19	0.0059	23,717	139	2,650
20	0.0043	21,429	91	1,828
21	0.0031	18,789	58	1,221
22	0.0022	15,797	35	780
23	0.0016	12,453	20	466
		$\sum$	68,600	439,687
		Mean generation time (T)		6.41

Supplementary Table 5 - Life table for Lake Superior *C. hoyi*.

Age (x)	Relative N (l_x)	Fecundity (m_x)	$l_x m_x$	$x l_x m_x$
3	1.0000	2,524	2,524	7,571
4	0.7247	2,791	2,023	8,090
5	0.5252	3,058	1,606	8,031
6	0.3806	3,324	1,265	7,592
7	0.2758	3,588	990	6,927
8	0.1999	3,848	769	6,153
9	0.1449	4,103	594	5,349
10	0.1050	4,352	457	4,569
11	0.0761	4,596	350	3,846
12	0.0551	4,833	266	3,197
13	0.0400	5,062	202	2,629
14	0.0290	5,285	153	2,142
15	0.0210	5,500	115	1,731
16	0.0152	5,707	87	1,389
17	0.0110	5,907	65	1,107
18	0.0080	6,098	49	877
19	0.0058	6,282	36	691
20	0.0042	6,459	27	542
21	0.0030	6,627	20	423
22	0.0022	6,789	15	329
23	0.0016	6,943	11	255
		$\sum$	11,624	73,438
		Mean generation time (T)		6.32

Supplementary Table 6 - Life table for Lake Superior *C. kiyi*.

Age (x)	Relative N (l_x)	Fecundity (m_x)	$l_x m_x$	$x l_x m_x$
4	1.0000	2,082	2,082	8,329
5	0.6145	2,330	1,432	7,158
6	0.3776	2,567	969	5,815
7	0.2320	2,794	648	4,538
8	0.1426	3,012	429	3,435
9	0.0876	3,220	282	2,539
10	0.0538	3,420	184	1,841
11	0.0331	3,611	119	1,314
12	0.0203	3,794	77	925
13	0.0125	3,970	50	644
14	0.0077	4,138	32	445
15	0.0047	4,299	20	304
16	0.0029	4,453	13	206
17	0.0018	4,601	8	139
18	0.0011	4,742	5	93
19	0.0007	4,878	3	62
20	0.0004	5,008	2	41
21	0.0003	5,132	1	27
22	0.0002	5,251	1	18
23	0.0001	5,366	1	12
		$\sum$	6,359	37,885
		Mean generation time (T)		5.96

References

1. Leslie P. The intrinsic rate of increase and the overlap of successive generations in a population of guillemots (Uria aalge Pont.). *Journal of Animal Ecology* **35**, 291-301 (1966).

2. Pratt T, Chong S. Contemporary life history characteristics of Lake Superior deepwater ciscoes. *Aquatic Ecosystem Health & Management* **15**, 322-332 (2012).

3. Yule DL*, et al.* Does Fecundity of Cisco Vary in the Upper Great Lakes? *North American Journal of Fisheries Management* **40**, 973-985 (2020).

4. Vinson MR*, et al.* Lake Superior Kiyi (Coregonus kiyi) reproductive biology. *Transactions of the American Fisheries Society* **n/a**, (2022).

5. Dryer WR, Beil J. Growth changes of the bloater (Coregonus hoyi) of the Apostle Islands region of Lake Superior. *Transactions of the American Fisheries Society* **97**, 146-158 (1968).
