# Supplementary material for: Origin of the Laurentian Great Lakes fish fauna through upward adaptive radiation cascade prior to the Last Glacial Maximum
Source: Commun Biol. 2024 Aug 12;7:978. doi: 10.1038/s42003-024-06503-z (PMC11319351; doi:10.1038/s42003-024-06503-z)
Supplement: Supplementary file 3 — Reporting Summary [file 42003_2024_6503_MOESM3_ESM.pdf]

nature portfolio

Corresponding author(s): Trevor J. Krabbenhoft

Last updated by author(s): May 28, 2024

Reporting Summary

Nature Portfolio wishes to improve the reproducibility of the work that we publish. This form provides structure for consistency and transparency in reporting. For further information on Nature Portfolio policies, see our Editorial Policies and the Editorial Policy Checklist.

Statistics

For all statistical analyses, confirm that the following items are present in the figure legend, table legend, main text, or Methods section.

n/a	Confirmed	
☐	☒	The exact sample size (*n*) for each experimental group/condition, given as a discrete number and unit of measurement
☐	☒	A statement on whether measurements were taken from distinct samples or whether the same sample was measured repeatedly
☒	☐	The statistical test(s) used AND whether they are one- or two-sided<br>*Only common tests should be described solely by name; describe more complex techniques in the Methods section.*
☒	☐	A description of all covariates tested
☒	☐	A description of any assumptions or corrections, such as tests of normality and adjustment for multiple comparisons
☒	☐	A full description of the statistical parameters including central tendency (e.g. means) or other basic estimates (e.g. regression coefficient) AND variation (e.g. standard deviation) or associated estimates of uncertainty (e.g. confidence intervals)
☒	☐	For null hypothesis testing, the test statistic (e.g. *F*, *t*, *r*) with confidence intervals, effect sizes, degrees of freedom and *P* value noted<br>*Give P values as exact values whenever suitable.*
☒	☐	For Bayesian analysis, information on the choice of priors and Markov chain Monte Carlo settings
☒	☐	For hierarchical and complex designs, identification of the appropriate level for tests and full reporting of outcomes
☒	☐	Estimates of effect sizes (e.g. Cohen's *d*, Pearson's *r*), indicating how they were calculated

Our web collection on statistics for biologists contains articles on many of the points above.

Software and code

Policy information about availability of computer code

Data collection	Coregonus artedi genome sequencing and assembly: Guppy v4.2.3+f90bd04, Trim Galore! v.0.6.0, Flye v2.8-b1674, bwa v0.7.17-r1198, Pilon v1.23,Purge Haplotigs v1.1.2, Kraken2 v.2.0.8-beta, Juicer v1.6,3D-DNA v18092, Chromonomer v1.13, BUSCO v5.1.2, RepeatModeler v2.0.1, blastx v2.9.0+, RepeatMasker v4.1.1. Coregonus sample population genetics and historical demography: TrimGalore! v0.6.6, bwa v0.7.17, BCFtools v1.15, VCFtools v0.1.17, R v4.3.0, vcfR v.1.10.0, adegenet v.2.1.3, IQ-Tree v2.2.2.6, ModelFinder v2.2.2.6, FigTree v.1.4.4, vcfutils.pl v0.1.17, PSMC v0.6.5-r67, PSMC utility 'psmc_plot.pl v0.6.5-r67', SMC++ v1.15.2, BCFTools v.0.1.19. Salvelinus namaycush population genetics and historical demography:bwa v0.7.17, BCFtools v.0.1.19, PSMC v0.6.5-r67, PSMC utility 'psmc_plot.pl v0.6.5-r67', SMC++ v1.15.2.
Data analysis	Scripts used for data analyses are available at https://github.com/KrabbenhoftLab/Coregonus_demography.

For manuscripts utilizing custom algorithms or software that are central to the research but not yet described in published literature, software must be made available to editors and reviewers. We strongly encourage code deposition in a community repository (e.g. GitHub). See the Nature Portfolio guidelines for submitting code & software for further information.

Data

Policy information about availability of data

All manuscripts must include a data availability statement. This statement should provide the following information, where applicable:

- Accession codes, unique identifiers, or web links for publicly available datasets
- A description of any restrictions on data availability
- For clinical datasets or third party data, please ensure that the statement adheres to our policy

The genome assembly and all sequence data are available under NCBI BioProjects PRJNA1062807 (Coregonus spp.) and PRJNA1077361 (Salvelinus namaycush). The numerical source data behind the graphs can be found in https://doi.org/10.5061/dryad.n02v6wx59.

Research involving human participants, their data, or biological material

Policy information about studies with human participants or human data. See also policy information about sex, gender (identity/presentation), and sexual orientation and race, ethnicity and racism.

Reporting on sex and gender	N/A
Reporting on race, ethnicity, or other socially relevant groupings	N/A
Population characteristics	N/A
Recruitment	N/A
Ethics oversight	N/A

Note that full information on the approval of the study protocol must also be provided in the manuscript.

Field-specific reporting

Please select the one below that is the best fit for your research. If you are not sure, read the appropriate sections before making your selection.

☐ Life sciences ☐ Behavioural & social sciences ☒ Ecological, evolutionary & environmental sciences

For a reference copy of the document with all sections, see nature.com/documents/nr-reporting-summary-flat.pdf

Ecological, evolutionary & environmental sciences study design

All studies must disclose on these points even when the disclosure is negative.

Study description	We sequenced and assembled the Coregonus artedi genome using Oxford Nanopore long reads and Illumina short reads. We also sampled and Illumina-sequenced 20 additional accessions to understand evolutionary relationships and historical demography within two Laurentian Great Lakes salmonid radiations.
Research sample	We sequenced and assembled the Coregonus artedi genome from a single individual that was collected from Lake Huron, USA, and raised in the USGS Great Lakes Center (Ann Arbor, Michigan, USA).
Sampling strategy	Coregonus and Salvelinus namayacush samples were selected based on availability of material with consideration to the biodiversity within the species complex within North America
Data collection	High molecular weight DNA for reference genome sequencing was obtained from flash frozen muscle tissue. DNA of resequenced Coregonus and Salvelinus was also obtained from flash frozen muscle tissue.
Timing and spatial scale	Coregonus samples from Lake Superior were collected between 5/29/2015 to 8/29/2015. Coregonus samples from Lake Nipigon were collected on 9/9/2018. The periodicity of collection was due to government agencies predetermined timing of sampling on large research vessels.
Data exclusions	None
Reproducibility	All data used in this study are publically available for reproducability.
Randomization	Not applicable for genome and sequence data
Blinding	Not applicable for genome and sequence data

Did the study involve field work? ☒ Yes ☐ No

Field work, collection and transport

Field conditions	NA
Location	Lake Superior (United States and Canada), Lake Nipigon (Canada)
Access & import/export	The following coauthors have all required research permits or permissions granted to collect the fish specimens in the field: (1) Dan Yule - State of Wisconsin Department of Natural Resources Scientific Collectors Permit (permit number SCP-NOR-073-0515) issued February 10, 2015, State of Michigan Department of Natural Resources Scientific Collectors Permit issued February 7, 2013.
Disturbance	Samples were subset from collections from fisheries management agencies.

Reporting for specific materials, systems and methods

We require information from authors about some types of materials, experimental systems and methods used in many studies. Here, indicate whether each material, system or method listed is relevant to your study. If you are not sure if a list item applies to your research, read the appropriate section before selecting a response.

Materials & experimental systems

n/a	Involved in the study
☒	☐ Antibodies
☒	☐ Eukaryotic cell lines
☒	☐ Palaeontology and archaeology
☐	☒ Animals and other organisms
☒	☐ Clinical data
☒	☐ Dual use research of concern
☒	☐ Plants

Methods

n/a	Involved in the study
☒	☐ ChIP-seq
☒	☐ Flow cytometry
☒	☐ MRI-based neuroimaging

Animals and other research organisms

Policy information about studies involving animals; ARRIVE guidelines recommended for reporting animal research, and Sex and Gender in Research

Laboratory animals	Study did not involve labratory animals
Wild animals	C. artedi, C. hoyi, and C. kiyi and S. namaycush were collected across Lake Superior. C. nigripinnis samples were collected from Lake Nipigon. Multiple gear types were used for collection.
Reporting on sex	No sex based analyses were completed.
Field-collected samples	Samples were collected as noted in the manuscript.
Ethics oversight	No ethical approval was required.

Note that full information on the approval of the study protocol must also be provided in the manuscript.

Plants

Seed stocks	N/A
Novel plant genotypes	N/A
Authentication	N/A
